# The effect of visually manipulating back size and morphology on back perception, body ownership, and attitudes towards self-capacity during a lifting task

**DOI:** 10.1007/s00426-021-01609-z

**Published:** 2021-11-02

**Authors:** Kristy Themelis, Natasha Ratcliffe, Tomohiko Nishigami, Benedict M. Wand, Roger Newport, Tasha R. Stanton

**Affiliations:** 1 School of Psychology, University of Nottingham, Nottingham, UK; 2 Department of Psychology, University of Warwick, Coventry, UK; 3 Department of Physical Therapy, Faculty of Health and Welfare, Prefectural University of Hiroshima, Hiroshima, Japan; 4 IIMPACT in Health, Allied Health and Human Performance, University of South Australia, Adelaide, SA Australia; 5 School of Physiotherapy, University of Notre Dame Australia, Fremantle, WA Australia; 6 School of Sport, Exercise and Health Sciences, Loughborough University, Loughborough, UK

## Abstract

Body re-sizing illusions can profoundly alter perception of our own body. We investigated whether creating the illusion of a muscled and fit-looking back (Strong) influenced perceived back size, body ownership, and attitudes towards self-capacity during a lifting task. Twenty-four healthy male volunteers performed a standardised lifting task while viewing real-time (delay < 20 ms) video of their own back through a head-mounted display under four different conditions (Normal size, Strong, Reshaped, Large; order randomised). The MIRAGE-mediated reality system was used to modify the shape, size, and morphology of the back. Participants were poor at recognizing the correct appearance of their back, for both implicit (perceived width of shoulders and hips) and explicit (questionnaire) measures of back size. Visual distortions of body shape (Reshaped condition) altered implicit back size measures. However, viewing a muscled back (Strong condition) did not result in a sense of agency or ownership and did not update implicit perception of the back. No conditions improved perceptions/attitudes of self-capacity (perceived back strength, perceived lifting confidence, and perceived back fitness). The results lend support for the importance of the embodiment of bodily changes to induce changes in perception. Further work is warranted to determine whether increased exposure to illusory changes would alter perceptions and attitudes towards self-capacity or whether different mechanisms are involved.

**Supplementary Information:**

The online version contains supplementary material available at 10.1007/s00426-021-01609-z.

## Introduction

The perception of the body in terms of its size, shape, and distinctive features is an intrinsic part of the body image and plays a fundamental role in our everyday lives (Ehrsson et al., 2005). Body perception is largely driven by sensory input from vision, proprioception, and touch relevant to the body part, from interoceptive signals, as well as through interactions with the external world (Azañón et al., 2016; Dijkerman & De Haan, 2007; Gallagher, 2005; Ionta et al., 2011). Different body parts inherently have different sensory and motor exposures. Consider the back and the hand, for example. Unlike the hand, we rarely have vision of our own back, we only perform gross general movements (versus fine grained movements), and we do not typically need to discriminate the precise location of where we are touched on our back (Catley et al., 2013). Because of limited direct visual access of the back, knowledge of its size and shape is important for navigating the body in space. Despite the importance of such knowledge, we know very little about how the back is represented (Taoka et al., 2016).

Even in very precisely represented areas, such as the hand, perceiving accurate body size is a complex process. For instance, when the hand is hidden from view and participants are asked to judge the location of the fingertip and the knuckle via pointing, people tend to overestimate hand width, and underestimate finger length—i.e. people perceive a short, fat hand (Longo & Haggard, 2010). This *implicit* perception of a short, fat hand is thought to occur as a result of tactile anisotropy—the tendency to perceive stimuli provided across the body as being farther apart than stimuli provided along the body—which reflects the orientation of tactile receptive fields and innervation density (Longo & Haggard, 2010; Ross & Murray, 2018). Yet, we can perform complex hand movements and interact with the environment, without error, which supports the existence of distinct implicit and explicit body representations (Longo, 2015; Mancini et al., 2014). However, recent observation supports the idea that motor responses are not necessarily based on more accurate hand size representations but are also influenced by interactions with the spatial environment as well as affordances and emotional content (Peviani et al., 2020; Peviani et al., 2021). Differences in implicit and explicit representations of body size may have relevance given that people with chronic back pain often have a distorted perception of their own back (Moseley, 2008; Nishigami et al., 2014; Wand et al., 2013) and perhaps one or both of these representations is altered. Little is known about the representations of the back even in healthy participants, which makes interpretations of findings in back pain populations difficult. Here we aim to explore both implicit and explicit back representations in healthy individuals.

It is well-established that our perception of our body is dynamic and can be manipulated. Indeed, multisensory body illusions can be used to alter body perception in terms of size and shape and have been shown to alter sensory (Schaefer et al., 2007) and motor (Dilena et al., 2019; Naito et al., 2016) representations for that body part. For example, previous work in people with and without painful hand osteoarthritis has shown that visuotactile illusions that alter the size of the viewed hand induce changes in perceived hand size that remain following illusion cessation (Gilpin et al., 2014). To date, research that demonstrates the possibilities of manipulating body size perception has largely focused on body parts that are highly and precisely represented within the cortex such as the hands, or, generally explored effects with changes to the whole body itself (Banakou et al., 2013). What is less understood is whether similarly influential modulation of perceived body size occurs when the representation of the body part is less precise, as occurs for body parts, such as the back, and whether more extreme departures from normal body morphology may also be possible in these areas.

Preliminary work has shown that visual exposure to extreme body types, by means of visual adaptation, can affect perceptual body image judgements (Ambroziak et al., 2019). Specifically, Hummel et al. (2013) found that after exposure to adapted thin and fat pictures of one’s own body, participants perceived their own body to be thinner or fatter, respectively, than their actual body size. Previous experimental research has also shown that illusions of body ownership not only elicit conscious perceptual changes of the body, but may also extend to implicit attitudes and behaviours based on socially and culturally derived presumptions (Banakou et al., 2013; Blanke et al., 2015; Maister et al., 2013, 2015). For example, Kilteni et al. (2013) showed that taking on a different virtual body results in both updated body representation and self-representation in terms of attitudes and behaviours. In that study, Caucasian individuals participated in a virtual West-African Djembe drumming session with a virtual body that substituted their own: either a male with a dark-skinned body wearing casual dress or a male with a light-skinned body wearing a formal suit. Participants who were given a virtual body representation of a casually dressed dark-skinned avatar exhibited higher variation and frequency of movement, than participants who were given a light-skinned formally dressed avatar (Kilteni et al., 2013). These results support the ability of virtual full body ownership illusions to induce substantial behavioural changes, dependent on the appearance of the embodied virtual avatar.

Thus, while there is clear evidence that changing the appearance of the body can lead to perceptual and behavioural changes, what remains unknown is whether such effects extend to alteration of our own self-perceptions. Here we aim to extend past work by exploring whether multisensory illusions that provide information about body properties—namely back strength—may influence attitudes about self-capacity. When we see a well-muscled individual, based on social presumptions, we might think that individual is ‘strong’. That is, we infer properties of strength and ability based on visual appearance. Previous studies have found a positive relationship between upper-body muscularity and actual upper-body strength (Candow & Chilibeck, 2005). Furthermore, upper-body muscularity positively influences self-perception of fighting ability in men, indicating that muscular men perceive their fighting ability as being greater, independent of how strong they actually are as measured by hand grip strength (Muñoz-Reyes et al., 2019). There is also evidence that an influence of size/musculature on perceived ability may extend to the back. In a proof-of-concept pilot study with two chronic back pain participants, Nishigami et al., (2019) used a multisensory illusion of the back that drastically altered body morphology (i.e. a muscled reshaped back). In a single case study, with altered body perception and negative back pain beliefs, the illusion led to a reduction in movement associated fear and a positive change in perceived lifting ability.

Therefore, we explored perception of the back in healthy individuals, with the aim to determine whether (1) people have accurate implicit and explicit perceptions of back size; (2) multisensory illusions designed to alter back morphology can modify perceptions of back size, shape, and embodiment in a way that is analogous to previously observed hand resizing; and (3) body morphological illusions can also alter perception and attitude towards self-capacity, such as perceived back strength and confidence. Specifically, we manipulated the back’s shape and its musculature, with the hypothesis that a perceived notion of a strong and fit back (visually apparent musculature and wide shoulders with narrow hips) might influence perceived abilities during a standardised lifting task. To isolate whether effects were specific to having a ‘Strong’ back, we also included various additional conditions that altered only size or shape of the back (no musculature changes). We hypothesised that due to imprecise sensory representations of the back and the limited direct visual experience, participants would be inaccurate at estimating the shape and morphology of the back for both implicit and explicit measures of back perception, and that illusions altering back morphology would influence back perception. Last, we hypothesised that an illusion of a stronger-looking back that is embodied would influence perceptions of back size and attitudes towards self-capacity, namely, perceived strength and confidence during a lifting task.

## Materials and methods

### Participants

Participants were recruited from staff and students at the local university via offline and online advertisement. Participants were also recruited via word of mouth and by snowball sampling. Participants were eligible if they were male, between 18 and 60 years old, fluent in written and spoken English, and able to provide informed consent. Male participants were specifically recruited because the illusion required an entirely bare trunk/back (i.e. could not have any clothing that covered any part of the back, such as a bra). Participants were not eligible if they had any ongoing medical complaint, had experienced any persistent pain in the past six-months, were experiencing any current pain, or had any significant visual impairment. The participants were blinded to the hypotheses of the study. This study was performed in line with the principles of the Declaration of Helsinki. This study was approved by the Human Research Ethics Committee (ID34289), University of South Australia and all participants provided written informed consent.

### Equipment

A head-mounted MIRAGE system was used to provide a visual illusion of the back (Nishigami et al., 2019; Preston & Newport, 2012). In brief, a customised LabVIEW program (National Instruments LabVIEW version 2010) manipulated video feedback of the back in real time (delay < 20 ms). Participants stood inside a testing area with matt black walls and wore black sleeves (from shoulder to fingertips) to frame their torso. A camera (Basler acA645) was situated on a tripod and placed 2 m behind the participant, with the captured video of the back displayed through a head-mounted display (HMD; Carl Zeiss, Cinemizer OLED) worn by the participants. The camera was positioned so that participants viewed their own back from the waist up. Participants were advised to imagine that they were looking at their own back—i.e. as though they were looking in double mirrors, one in front (view in goggles) that reflected the image of their own back from a larger mirror placed behind them. Two reflective markers were attached to the participant’s back at the level of the T3 and L5 vertebrae (identified via palpation). The customised LabVIEW software manipulated the back image in real time and included altering back size and/or merging an overlay of a generic, muscled back (of the same size and shape) onto the viewed body. The program specifically used the markers to track movements of the back and stabilise the merged back image during movement.

### Experimental conditions

**Fig. 1:**
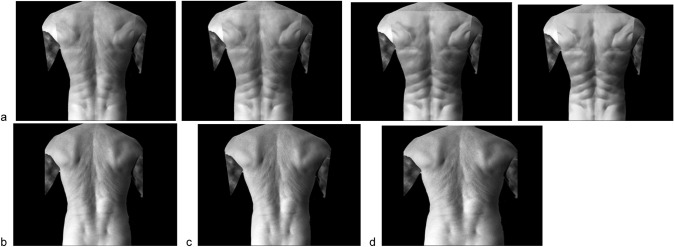
Illustration of testing conditions. **a** Strong back illusion, showing the gradual visual morphing of normal to muscled back (shoulders 25% wider and hips 25% narrower, final image showing the illusion test condition); **b** Normal (veridical) back condition; **c** Reshaped condition (shoulders 25% wider and hips 25% narrower); **d** Large condition (shoulder and hips 25% wider)

All participants completed four experimental conditions (Strong, Normal, Reshaped, Large) in a randomised order (Fig. 1). In the Strong condition (Fig. 1a), an illusion of a muscled, fit back was created by merging an image of a generic muscled back onto the participants own back, while also broadening the shoulders by 25%, and narrowing the waist by 25%. In the Normal condition (Fig. 1b), participants viewed their back without any visual manipulation. This condition served to control for the effect of simple vision of the back on perception of the back, In the Reshaped condition (Fig. 1c), the shoulders were also broadened by 25% and the waist narrowed by 25%, (i.e. creating a fit body shape) but without overlaying an image of a muscled back. In the Large condition (Fig. 1d), both the shoulders and waist were increased by 25%. This condition served to determine whether increased size of the back (versus increased ‘fit’ body shape) was most influential on perception during a lifting task.

### Lifting task

In all conditions, participants lifted a box with a set weight of 6.6 kg and held it in a stoop-like posture (straight legs and slightly bent forward from the waist) for 90 s. During each condition participants squeezed their shoulder blades together every 20 s (0 s, 20 s, 40 s, 60 s, 80 s). This method was chosen because temporal synchrony between seen and felt movement has been shown to be important drivers of embodiment (Ratcliffe & Newport, 2017). Our pilot data showed that a 6.6 kg weight was reported to feel like a light to medium weight without eliciting back pain.

### Outcome measures

#### Explicit back perception

Explicit back perception was explored in two ways. First, it was formally evaluated at the end of experimental testing by asking participants to identify in which experimental condition they perceived that they were looking at the correct appearance of their back. We calculated the proportion of participants that correctly identified the Normal condition. This assessment was only performed after all conditions were completed to minimise the potential for immediate unblinding and for response-shift bias (Howard, 1980) (i.e. the ratings for one condition [e.g. this condition was my back] influencing subsequent ratings [e.g. the next condition could not be my back, because I already rated the previous one as my own]).

In addition, explicit back perception was assessed at baseline via the Fremantle Back Awareness Questionnaire (FreBAQ) (Wand et al., 2014), which describes various features related to back perception (i.e. including perceived size). This questionnaire shows adequate internal consistency and test–retest reliability and discriminant validity, i.e. the ability to discriminate between individuals with and without lower back pain (Wand et al., 2016). Higher scores represent higher levels of altered/disturbed back perception (Schäfer et al., 2021; Wand et al., 2016).

#### Implicit back perception

**Fig. 2:**
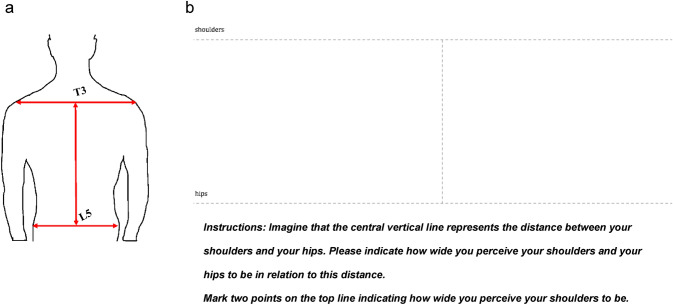
Back-size estimation task. **a** Illustration of the back measurements taken and **b** example of the back-size estimation task. **a** Measurements were taken from the most prominent bone at the base of the neck at the 3rd thoracic vertebra (T3) to the natural waistline at the 5th lumbar segment (L5). The shoulders were measured from the outside of one shoulder to the other, crossing T3 and the hips were measured from the outside on one hip to the other crossing L5. **b** Participants were asked to indicate how wide they perceived their shoulders and their hips to be in relation to a central vertical line which was printed at 10 cm on a blank piece of paper. Participants were instructed to mark two points on the top line and two points on the bottom-line indicating shoulder width and hip width, respectively

Implicit back perception was assessed using a back-size estimation task (Fig. 2). Specifically, a template of a cross was used (Fig. 2a), with a vertical line of 10 cm serving as an anchor, representing the length from T3 (roughly at the top of the shoulder blades) to L5 (the curve just above the buttocks). Horizontal lines spanning the width of the A4 paper represented the width of the shoulders and the hips (Fig. 2b). Participants were asked to estimate the relative location of their shoulders and hip with respect to the vertical anchor line (i.e. “If the length of the vertical line represents the length of your own back, how wide do you perceive your shoulders/hips to be”?). We referred participants to the actual measurements taken (T3–L5) as a reminder of the exact locations we were asking them to estimate. Participants were instructed to mark two points on the top horizontal line to indicate how wide they perceived their shoulders to be (outer edge of left shoulder, outer edge of right shoulder) and two points on the bottom horizontal line to indicate how wide they perceived their hips to be (outer edge of left hip, outer edge of right hip).

To investigate whether people have accurate perceptions of back size, perceived body widths (shoulder and hip) at baseline, in which participants lifted the box and viewed their back through the HMD, were compared to actual body widths. To do this, the actual length from T3 to L5 in relation to the vertical anchor line on the implicit back template was first calculated (actual length/vertical anchor = proportional length). Next, the estimated shoulder and hip width was calculated in relation to the proportional length (estimated shoulder width*proportional length = normalised perceived body width). The shoulder/hip ratio was calculated after each condition by dividing the estimated shoulder width by the estimated hip width (shoulder/hip ratio = shoulder width/hip width). Higher values represent the perception that the width of the shoulders is larger than the width of the hips (i.e. an enhanced inverted triangle body shape).

In order to investigate whether altered back morphology can modify perceptions of size and shape, Shoulder width percent (%) error and Hip width percent (%) error were also calculated. Specifically, the estimated shoulder and hip width after each experimental condition was calculated as the difference from respective body width estimation at baseline, and then expressed as a percent (%) error of the baseline body width (%error body width = [estimated body width – baseline body width]/baseline body width *100).

#### Embodiment

Back embodiment was assessed via a 10-item modified embodiment questionnaire (Nishigami et al., 2019). The questionnaire assessed three categories of embodiment: ownership, feelings towards their own back, and agency (Table 1). It also consisted of one control question. Using a 7-point rating scale, participants were asked to rate their agreement to each statement after each condition (− 3 = strongly disagree; + 3 = strongly agree). Two of the items “I felt as though the back I was looking at did not belong to me” and “I felt as though someone else was causing the movement of the back that I was looking at” are reverse scored. The average score was calculated separately for each category.

**Table 1:** Items used for the embodiment questionnaire

Q	Item	Category
1.	I felt as though the back I was looking at was part of my body	Ownership
2.	I felt as though I was looking at my own back	
3.	I felt as though the back I was looking at did not belong to me	
4.	I liked the way my back looked	Feelings towards own back
5.	My back felt more supported than usual	
6.	I would like my back to stay the way that I saw it	
7.	I felt as though I was in control of the back I was looking at	Agency
8.	Whenever I moved, I expected the back I was looking at to move in the same way	
9.	I felt as though someone else was causing the movement of the back that I was looking at	
10.	I felt as though my back had disappeared	Control

#### Perceptions and attitudes related to self-capacity of the back

Perceptions of back strength, confidence in the back, back fitness, and heaviness of the lifted box were assessed using a 100-mm visual analogue scale (VAS) immediately after task completion (Table 2). This aimed to capture participants’ perceptions of their own ability related to their back with respect to the lifting task. Perceived back strength was assessed by asking participants how strong their back felt after each condition with anchors of “not strong at all” to “strongest imaginable”. For perceived confidence, participants were asked to rate how confident they would be in their ability to lift something light or something heavy from the floor, with anchors of “not confident at all” to “most confident imaginable”. For perceived back fitness, participants were asked how fit their back felt at the moment responding on a scale with anchors of “not fit at all” to “fittest imaginable”. Perceived heaviness of the box was assessed by asking participants about how light or heavy the box felt in the lifting task measured on a 0–100 mm VAS with anchors of “extremely light” to “extremely heavy”. Last, in case the lifting did elicit pain or discomfort, participants rated how much pain they were experiencing after each condition, measured on a 0–100-mm VAS with anchors of “no pain at all” to “most pain imaginable”.

**Table 2:** Perception and attitude items related to self-capacity of the back

Q	Question
1.	How strong does your back feel at the moment?
2.	If you had to lift something light from the floor, how confident would you be in your ability to do this?
3.	If you had to lift something heavy from the floor, how confident would you be in your ability to do this?
4.	How fit does your back feel at the moment?
5.	How heavy did the box you were just lifting felt to you?
6.	How would you rate the pain in your back during the lifting task?

### Procedure

Participants first provided demographic information and completed the FreBAQ questionnaire. To allow comparison of perceived back size to true back size, measures of actual back size were taken. Thus, in all participants, the length of the back was measured (using a tape measure) from the 3rd thoracic vertebra (T3; roughly at the top of the shoulder blades) to the natural waistline at the 5th lumbar vertebra (L5). The shoulders were measured from the lateral aspect of each shoulder, spanning T3, and the hips were measured from the lateral aspect of each hip, spanning L5.

Prior to commencement of the experimental conditions, two white paper markers were attached to participants’ backs between T3 and L5 using dressing tape. Black draping cloths were used to cover participants’ legs (from the waist down), and the black sleeves were used to cover their arms. Following this, participants viewed their own back from the waist up through a head-mounted display (HMD) connected to a camera positioned behind their body. They then lifted the box and acclimatized themselves to the experimental set-up, at which point a baseline measure of implicit back perception was taken. The 4 experimental conditions paired with the lifting task were undertaken in a randomised order and using the methods described above. After each condition, measures of embodiment (Table 1) and measures of implicit back perception, perceived back strength, perceived confidence, perceived fitness, and pain intensity were taken (Table 2). It was emphasised that the implicit questionnaire was referring to how their back felt (not what they had seen). The order of the embodiment questionnaire items was randomised for each condition. Participants removed the HMD between each condition for a 1-min break period. After all experimental conditions had been completed, participants were asked to identify, in which condition, the seen real-time video of their back was accurate (i.e. their back at the correct size/shape, not a manipulated image).

### Statistical analysis

#### Power

Twenty-four participants were recruited to allow detection of a moderate effect (Cohen’s *f* = 0.25) given 1 group, 4 test conditions (see details below), 80% power, a correlation of 0.5 between conditions, and an alpha of 0.05. The decision of powering for a moderate effect was made to achieve a balance between practicality and relevant effect detection.

#### Back perception

Data were assessed for normality using visual inspection and Shapiro–Wilk statistic and the assumption of normality was met for all dependent variables. To investigate whether participants held accurate implicit perceptions of back size, we conducted three separate paired samples *t*-tests between actual and baseline measurements of shoulder width, hip width, and shoulder/hip ratio as outcome measures. One extreme outlier was identified (3 box-lengths from the edge of a boxplot) and was removed from all analyses involving these means. To examine the differences in proportion of experimental conditions reported to be the actual back (explicit back perception), a *χ*^2^ test was conducted. To investigate the effect of illusions to alter implicit back perception we conducted three separate repeated measures ANOVAs with Shoulder % error, Hip % error, and shoulder/hip ratio as outcome measure taken after each experimental condition. Several outliers were detected—these were identified as data points more than 1.5 box-lengths from the edge of a boxplot. Inspection of their values did not reveal them to be extreme (more than 3 box-lengths) and thus they were kept in the analyses. Omnibus *F*-tests were followed by post-hoc tests with Bonferroni correction for multiple comparisons. Sphericity was assessed and when the assumption was not verified, the Greenhouse–Geisser correction was applied.

#### Embodiment and attitudes towards self-capacity

As the data were not normally distributed (as per visual inspection and Shapiro–Wilk statistic), non-parametric measurement for related samples was used. Friedman’s two-way analyses of variance by ranks and post-hoc tests were performed to identify differences among the means of the embodiment and attitude variables between conditions. Pairwise comparison post-hoc tests were performed with a Bonferroni correction for multiple comparisons. Adjusted *p* values are reported.

## Results

### Demographic data

The participant characteristics are presented in Table 3. Body Mass Index (BMI) for the total sample was within the range typically indicative for a healthy body weight (Mean = 24, SD = 4).

**Table 3:** Participant demographic and baseline information

Demographics	Mean (SD)
Age (years, SD)	24 (4.95)
Height (cm, SD)	178 (6.6)
Weight (kg, SD)	77.7 (13.6)
BMI (kg/m^2^)	24 (4)
Current pain intensity (0–100)	15 (34.9)
FreBAQ score	4.38 (4.61)

### Aim one: do people have accurate perceptions of back size?

#### Implicit back perception

Paired sample *t*-tests showed no significant difference in estimated shoulder width at baseline (*M* = 40.88, SD = 14.01) compared to actual shoulder width (*M* = 46.77, SD = 3.33), *t* (21) = 1.951, *p* = 0.065, *d* = 0.42. Similarly, there was no significant difference in estimated hip width at baseline (*M* = 31.25, SD = 10.18) compared to actual hip width (*M* = 32.73, SD = 5.38), *t*(21) = 0.660, *p* = 0.516, *d* = 0.14, but a significantly lower estimated shoulder/hip ratio at baseline (*M* = 1.31, SD = 0.18) compared to actual shoulder/hip ratio (*M* = 1.45, *SD* = 0.16), a mean difference of 0.14, *t*(21) = 2.97, *p* = 0.007, and *d* = 0.63. This equated to an average under-estimation of 13.16% (SD = − 23.79), meaning that while they perceived their shoulders as wider than their hips, it was significantly less so than their actual shoulder to hip width ratio.

#### Explicit back perception

The Pearson’s *χ*^2^ test showed significant differences in the proportion of experimental conditions being perceived as the actual back, *X*^2^(3) = 10.217, *p* = 0.017. Of the 23 participants that had indicated under which condition they thought they were looking at their actual back, only 13% answered correctly. Overall, 43.5% thought they viewed their real back in the Reshaped condition, 39% in the Large condition, and 4% in the Strong back condition.

### Aim two: can multisensory illusions alter perceptions of back size, shape, and embodiment?

#### Effect of condition on back perception

**Fig. 3:**
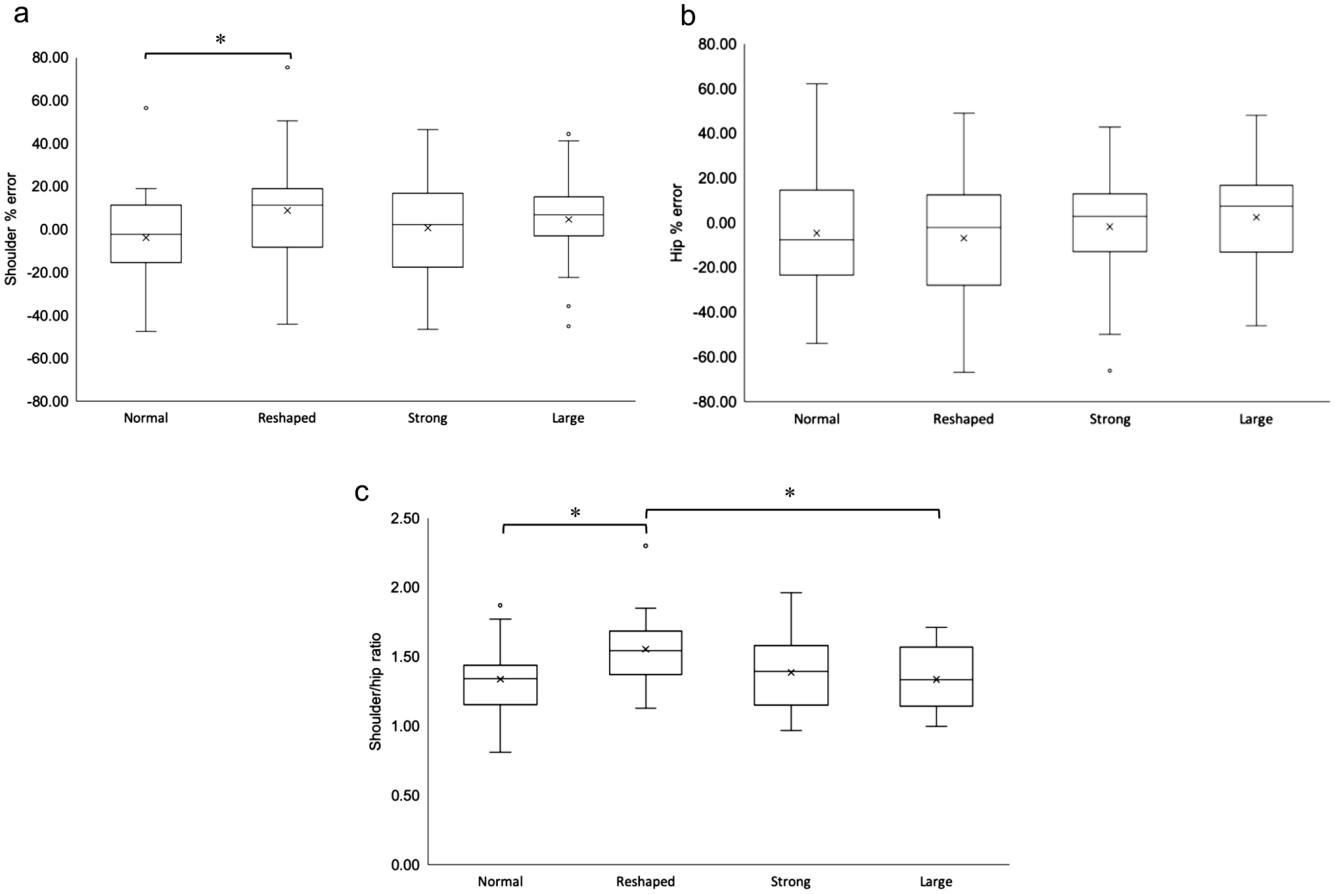
Box-whisker plots showing measures of implicit back perception. **a** shoulder % error and **b** hip % error were expressed as the difference between the estimated body width and the body width estimation at baseline, as a percent (%) error of the baseline body width (percent % error body width = (estimated body width-baseline body width)/baseline body width *100). **c** Shoulder/hip ratio was calculated by dividing the estimated shoulder width by hip width. A larger number represents an enhanced inverted triangle body shape. Solid horizontal lines indicate the median and the 25th–75th interquartile range level. A one-way repeated measures ANOVA with Bonferroni correction for pairwise comparisons was performed between conditions, and * indicates a statistically significant difference. Values outside the whiskers are conventionally called ‘outliers’ with values less than 1.5 box-lengths from the edge of the box and are shown by (º)

The repeated measures ANOVA on the Shoulder % error ratio after the experimental conditions showed a significant effect of condition, *F* (3, 66) = 4.447, *p* = 0.007, partial *η*^2^ = 0.168. Post-hoc analyses with a Bonferroni adjustment revealed that Shoulder % error was significantly higher in the Reshaped condition (*M* = 8.79%, SE = 5.85%) compared to the Normal condition (*M* = − 3.83%, SE = 4.75%), a statistically significant mean increase of 12.62%, SE = 3.261%, 95% CI [3.16, 22.07], p = 0.005, with no significant differences between any of the other conditions (All *M* > 8.09%, *p* < 0.145) (Fig. 3a).

The repeated measures ANOVA on the Hip % error ratio after the experimental conditions showed no significant effect of condition, *F* (3, 66) = 1.52, *p* = 0.219, partial *η*^2^ = 0.064 (Fig. 3b).

The repeated measures ANOVA on the shoulder/hip ratio after the experimental conditions showed a significant effect of condition, *F* (2.313,53.206) = 7.899, *p* = 0.001, partial *η*^2^ = 0.256. Post-hoc analyses with a Bonferroni adjustment revealed that body ratio was significantly higher in the Reshaped condition (*M* = 1.56, SE = 0.051) compared to the Normal condition (*M* = 1.34, SE = 0.045), a statistically significant mean increase of 0.218, SE = 0.047, 95% CI [0.081, 0.354], *p* = 0.001. Body ratio in the Reshaped condition was also significantly higher compared to the Large condition (*M* = 1.34, SE = 0.046), a statistically significant mean increase of 0.218, SE = 0.047, 95% CI [0.082, 0.354], *p* = 0.001, with no significant differences between any of the other conditions (All *M* > 169, *p* < 0.096) (Fig. 3c). This supports an alteration in implicit back perception in the Reshaped condition, consistent with the direction of manipulation.

#### Effect of condition on embodiment

**Fig. 4:**
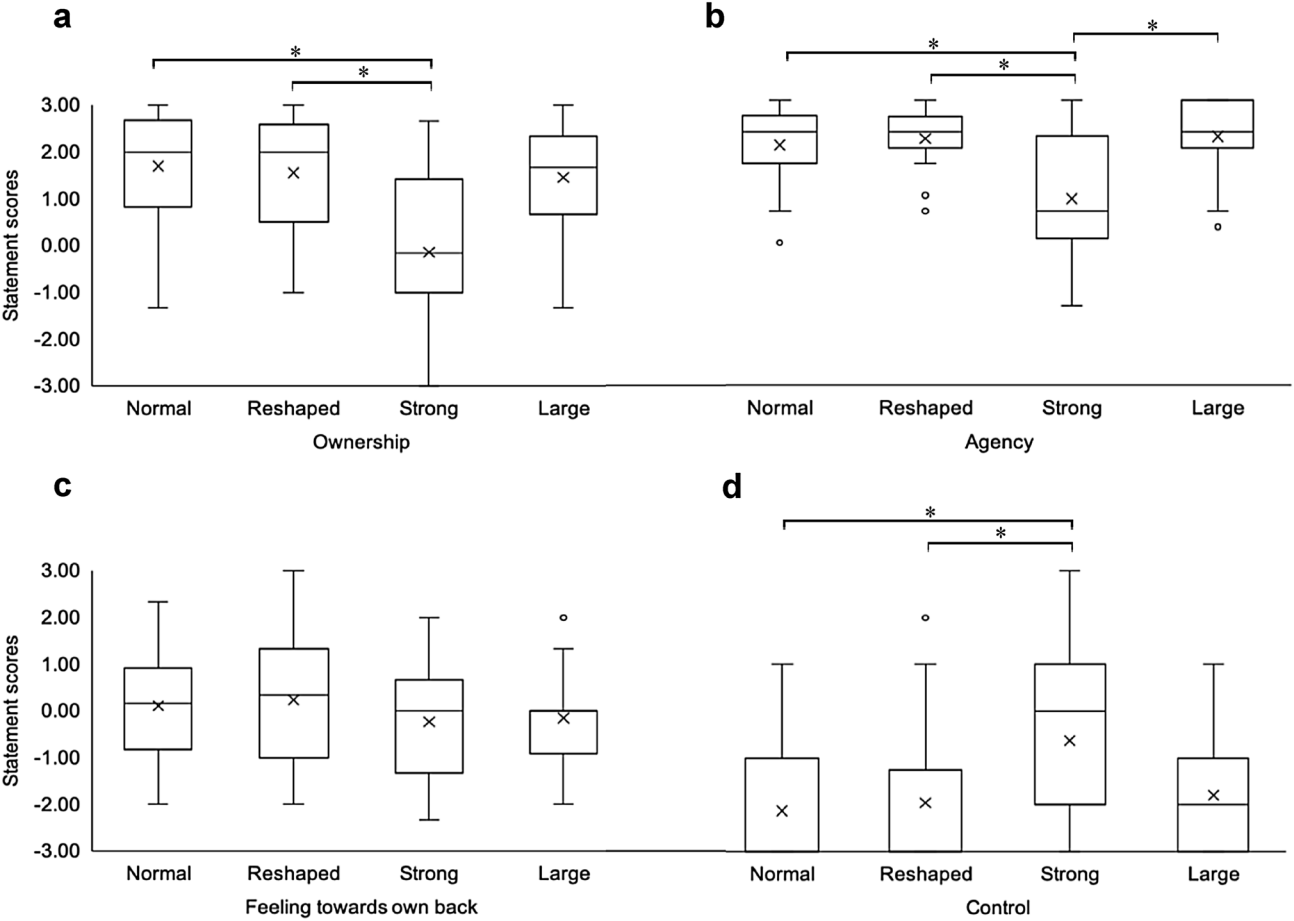
Box-whisker plots showing answers to the separate components on the embodiment. Questionnaire. Responses were measured using a 7-point Likert scale ranging from − 3 to + 3 with − 3 corresponding to ‘totally disagree’ and + 3 corresponding to ‘totally agree’. The items **a** Ownership, **b** agency, **c** feelings towards own back was set up to measure the illusion experience, whereas the **d** control item was set up to control for suggestibility and compliance. Solid horizontal lines indicate the median and the 25th–75th interquartile range level. A Friedman test and post-hoc tests with Bonferroni correction for pairwise comparisons were performed between conditions, and * indicates a statistically significant difference. Values outside the whiskers are conventionally called ‘outliers’ and are shown by (º)

Separate Friedman tests were conducted to determine if there were differences in ownership, agency, and positive feelings towards the back on the mean scores of the items in each subcategory (Table 1). Ownership ratings were significantly different between conditions, *X*^2^(3) = 16.466, *p* < 0.001, (Fig. 4a). Post-hoc analyses revealed statistically significant differences in ownership ratings between Strong (*Mdn* = *− 0.1*7) and Normal (*Mdn* = 2.0) (*p* = 0.001) and the Strong and Reshaped (*Mdn* = 2.0*)* (*p* = 0.037) conditions. None of the other pairwise comparisons were significant (Supplementary Table 1).

Similarly, Agency ratings were statistically significantly different between conditions, *X*^2^(3) = 21.802, *p* < 0.001, (Fig. 4b). Post-hoc analyses revealed a similar pattern to the ownership ratings with significant differences in median agency ratings between Strong (*Mdn* = 0.67) and Normal (*Mdn* = 2.33) (*p* = 0.031), Strong and Reshaped (*Mdn* = 2.33) (*p* = 0.022), and additionally between the Strong and Large (*Mdn* = 2.33) (*p* < 0.001) conditions. None of the other pairwise comparisons were significant (Supplementary Table 1).

In contrast, feelings towards one’s own back did not differ between conditions, *X*^2^(3) = 0.653, *p* = 0.884, (Fig. 4c). The ratings for the control question “I felt as though my back had disappeared”, were significantly different between conditions, *X*^2^(3) = 23.036, *p* < 0.001, (Fig. 4d). Post-hoc analyses revealed significantly higher median control scores in the Strong condition (*Mdn* = 0.0) compared to Normal (*Mdn* = *− *3) (*p* = 0.007) and Strong compared to Reshaped (*Mdn* = *− *3) (*p* = 0.015), but not between the other conditions (Supplementary Table 1).

### Aim 3: effect of condition on perceptions of the back and attitudes of self-capacity

Separate Friedman tests were conducted to determine if there were differences in perceived back strength, perceived confidence, perceived fitness, perceived heaviness, and pain between the different conditions (Supplementary Table 2). Median perceived confidence ratings in lifting a light object were not statistically significantly different between conditions, *X*^2^(3) = 5.323, *p* = 0.150. Similarly, perceived confidence in lifting a heavy object from the floor did not differ between conditions, *X*^2^(3) = 2.807, *p* = 0.422, nor did perceived back fitness, *X*^2^(3) = 3.717, *p* = 0.294, perceived back strength, *X*^2^(3) = 5.269, *p* = 0.153, perceived heaviness of the box, *X*^2^(3) = 2.077, *p* = 0.557, or pain ratings, *X*^2^(3) = 1.507, *p* = 0.681.

## Discussion

This study aimed to investigate whether people hold accurate size perceptions of the back, a body part rarely seen directly, and if perceptions of size can be manipulated using multisensory illusions. Additionally, this study aimed to evaluate whether these illusions can influence attitudes towards self-capacity, such as perceived strength and confidence. In line with our predictions, we found that participants have an inaccurate perception of the size and shape of their back, both measured on an implicit template task and when explicitly asked. Moreover, we found that perceptions of back size can be altered by morphological body illusions, but this appeared specific to the condition. In particular, we found that the Reshaped back condition significantly altered perceived body shape in a manner consistent with the direction of the illusion (wider shoulders and narrower waist). However, the Strong back illusion did not alter perceived body shape, despite the same body size reshaping occurring in both the Strong and Reshaping condition, potentially due to failure to induce reliable agency and ownership that occurred during the Strong condition. Against our hypotheses, none of the illusions influenced perceived strength, confidence, back fitness, or perceived box weight during the lifting task. The results suggest that back representation in healthy volunteers can be modified despite relatively large departures from its natural size when the underlying existing/natural features of the back are retained. Further, our results raise the possibility that when the viewed back is replaced by altered morphology (Strong, muscled back), embodiment of the manipulated image may be required in order to update back representation.

Our findings show that people have altered explicit and implicit representations of their backs that differ from each other and from actual back size. Explicit judgements were found to typically overestimate the hip–shoulder ratio. Specifically, most participants perceived the Reshaped condition as being indicative of the real appearance of their back (shoulders *wider* and hips *narrower* than they actually are). In contrast, implicit judgements were found to underestimate the hip–shoulder ratio. That is, while participants did perceive their shoulders to be wider than their hips (inverted triangle consistent with the Reshaped condition), the perceived ratio was smaller than veridical measurements. The same has been shown for other and more ‘familiar’ body parts, with the implicit representation of the hand being highly inaccurate and distorted (Longo et al., 2015). Past work has also shown an overestimation of implicit representation of shoulder width (via shoulder width-to-height ratio) in healthy individuals (Fuentes et al., 2013). If the same were true of our sample, it would suggest that our findings of a reduced shoulder to hip ratio (versus actual) may be driven by hip width changes relative to the shoulder. This explanation is supported by the magnitude of the effect size of the difference in estimated shoulder width at Baseline compared to actual shoulder width at 0.42 and a significant increase shoulder % error after the Reshaped condition compared to Normal. Future work appears warranted to further explore these differences.

That participants were consistently incorrect in explicitly identifying which test condition showed the correct appearance of their back (veridical, only 3/23 were correct), supports the idea that visual representation of the back is not precise and highlights just how unfamiliar we are with it. Further, these findings provide support for the contention that explicit representations of a body part that is not commonly seen likely differ from those of a body part that is commonly seen. For example, previous studies evaluating more familiar, commonly seen body parts show that individuals are accurate in making explicit judgements of the correct ratio of their hands (Longo & Haggard, 2010) and hip width/height (Fuentes et al., 2013). However, that we asked participants to identify their back from a range of differently shaped and/or modified backs based on their real back, and past studies relied upon participants identifying body shapes from pictures or templates of a third person, may also influence the differences seen (Longo & Haggard, 2012). Regardless, that participants did not report liking their back in the Reshaped condition more so than during the other conditions suggests that the alterations in explicit perceived back size seen here are not driven by a desire to have that back shape (wide shoulders, narrow hips). Additionally, that none of the participants exhibited a disordered mental representation of the back as suggested by the below average score on the FreBAQ questionnaire (Schäfer et al., 2021; Wand et al., 2016), suggests that the observed inaccuracies of explicit back size are likely an inherent feature of the healthy somatosensory system.

A key finding of our study is that the Reshaping illusion leads to an altered perception of body shape. This finding indicates that implicit body representation is flexible and that participants can take on an altered sized back as their own, despite relatively large departures from normal size and a complete lack of explicit self-recognition. While in this illusion the back is viewed in a different location than it actually is, participants were advised to imagine it from a first-person perspective. That is, they were asked to imagine that they were looking at their own back via double mirrors, with one in front (the view in the goggles), reflecting the image of their own back from a larger mirror placed behind them. Previous work has demonstrated that perceiving a mannequin as mirrored when viewing it in a third-person perspective results in similar levels of ownership as when viewing the mannequin in a first-person perspective (Preston et al., 2015). Indeed, the Reshaped condition resulted in a significantly larger shoulder/hip ratio (indicative of a body with broader shoulders and narrower waist) than both the Normal condition and the Large condition, and yet maintained ownership and agency in participants. However, that the Strong condition did not result in an altered perception of the back (shoulder/hip ratio) compared with the Normal condition, despite identical back resizing as the Reshaped condition is an important finding. The primary difference between the Reshaped and the Strong condition was the degree of embodiment of the illusion. That is, in the Strong condition, despite visual and tactile/motor synchrony, participants did not feel as though they had ownership or agency over the viewed back. In contrast, participants reported high levels of ownership and agency of the viewed back in the Reshaped condition. Thus, that the Reshaped condition altered back perception, but the Strong condition did not, despite identical changes in back size (wide shoulder, narrow hips/waist), therefore suggests a role of ownership in updating back perception. Taken together, our results suggest that while the representation of the body can be updated via multisensory illusions, it is essential that the manipulated body is felt to be the participant’s own and/or belong to the participant.

Contrary to our hypothesis, the appearance of a strong, muscled back did not translate to an increase in perceived back strength or ability. This appears inconsistent with past work which has shown that an objective visual measurement of muscularity, but not strength per se, predicts self-perceived fighting ability in men (Muñoz-Reyes et al., 2019). However, as mentioned above, our results may not be overly surprising given the lack of embodiment seen for the Strong illusion condition. Past work has shown that when embodiment is removed (e.g. using asynchronous sensory stimuli to induce loss of ownership and agency), changes in attitudes related to the body do not occur (Banakou et al., 2013). Thus, the loss of ownership and agency during the Strong illusion condition precludes the ability to conclude that viewed morphological changes do not influence attitudes towards self-capacity. Rather, we can state that viewed morphological changes that are not embodied do not influence such attitudes. Indeed, past work in a participant with chronic back pain showed that embodying the Strong illusion did result in shifts in attitudes towards self-capacity (e.g. increased confidence in lifting) (Nishigami et al., 2019).

While not a primary question of our study, it is interesting to note that despite embodiment of the other illusions, changes in perceived body strength did not occur when the back was made to look fitter (Reshaped condition) or bigger (Large condition). These findings raise the possibility that altering back size alone does not influence attitudes related to self-capacity. However, it is also interesting to consider that perceptions of strength or lifting ability, as measured here, may involve a cognitive component: predicting whether an action can be safely undertaken (and the ease of this) given the present assessment of body state. Given the widely held societal assumptions that lifting with a rounded back is unsafe (Caneiro et al., 2018) and that all lifts performed here were with a rounded back, it raises the possibility that cognitive features may have played a role, possibly preventing any shifts in perceived capacity. Future work undertaking the task in a straight back lifting posture may be warranted to allow the greatest possibility for multisensory changes to shift attitudes towards self-capacity.

It is interesting to consider why the Strong back condition resulted in a significant loss of both ownership and agency, and that this did not occur in the Reshaped condition with similar body size changes (Shoulders 25% wider and hips 25% narrower) and despite similar embodiment procedures undertaken in all conditions. First, our results support the presence of retained ownership and agency despite large visual distortion as has been shown previously (Ratcliffe & Newport, 2017). Second, it is possible that perceptual changes (i.e. size) may be more likely to maintain a sense of ownership and agency than alterations in physical attributes, that may be more readily identifiable as unrealistic, such as an overly muscled back as used here. Such an idea is consistent with findings from psycho-physiological studies suggesting that conflictual information over a body part can result in the loss of ownership and feeling of disownership over the actual body part (Barnsley et al., 2011; Moseley et al., 2008; Newport & Gilpin, 2011). Further, Tsakiris and Haggard (2005) demonstrated that ownership over the artificial hand only occurs when the object is a realistic-looking rubber limb, suggesting that the Strong back may be too morphologically and functionally different to embody. Indeed, participants reported feeling less like their back was present in the Strong condition, suggesting it ceased to be ‘back-like’ altogether. However, ownership has been reported over non-corporeal objects (Cordier et al., 2020; Ma & Hommel, 2015) or even empty space (Guterstam et al., 2013).

Third, it is well established (e.g. Botvinick & Cohen, 1998) that embodiment of an artificial limb or body part is the result of the integration of synchronous visual, tactile, and proprioceptive input. In the current study, the muscled back image was slowly morphed over the real back view and temporal and spatial synchronicity between vision, touch, and movement was attempted. Specifically, an embodiment procedure of touching and moving the viewed back was undertaken, followed by viewed movement of the back throughout the lifting task. However, it is possible that the overlaid strong muscles were viewed as a static image that was incongruent with movement. That is, the viewed overlaid muscles did not move as would be expected with self-initiated movement (e.g. flexing and relaxing with movements). Therefore, it is possible that incongruence between viewed and felt movement was induced in the Strong condition, potentially inducing a loss of ownership and potentially even agency. Together, these findings suggest that further testing of a muscled illusion is warranted, particularly exploring ways to promote maintained embodiment, such as increasing animation and life-like nature of the muscled overlay.

Finally, findings indicated that participants experienced low levels of pain after each condition, which is not uncommon following sustained muscle contraction (Minetto et al., 2013). Notably, pain ratings did not significantly differ compared to baseline or between conditions and the mean pain rating after each condition did not exceed 20/100 on the NRS, suggesting that pain did not contribute to any of the observed effects.

The current study had several limitations. Here we used an image of a very muscled back, consistent with a body builder (e.g. all back muscles visually apparent and well defined). Future work may be warranted to evaluate a less extreme muscled version. Additionally, it might be useful to determine the gradient of muscular change at which loss of ownership occurs; this would serve as a useful control condition. Furthermore, we did not investigate whether participants experienced any underlying issues of perceptual body image more generally (not just related to the back), for instance, eating disorders relating to the width of the lower abdomen and how this might influence their response at baseline.

The nature of our sample, collecting data in only young male participants limits the extent to which the findings might generalise. Although evidence suggests that there are no sex differences in the response to multisensory illusions (Petkova & Ehrsson, 2008) or more specifically, no sex differences in perceptual or emotional responses to illusory changes in body size (Preston & Ehrsson, 2014), we cannot exclude that assumptions of body strength and/or ability might differ between sexes. We solely recruited men both for practical reasons, but also due to the fact that muscularity is more universally viewed as a positive feature and a physical goal in men, whereas muscularity is sometimes viewed more negatively in women, for whom thinness may be prioritised (Grossbard et al., 2009). Hence, we aimed to evaluate men to provide a greater likelihood that a muscular back illusion would not invoke a negative body image. Regardless, further investigations are needed to better characterize pre-defined assumptions of strength and ability as well as issues of perceptual body image at baseline, testing across the spectrum of gender identity, and across different age groups, and how this might influence back perception, strength, and ability.

## Conclusions

Underlying implicit and explicit perceptions of back shape are inaccurate. Implicit perception can be modified by the embodiment of a visually altered back shape using multisensory illusion techniques. In contrast, visually modifying the appearance of the back so that it was excessively muscled resulted in a failure to induce a sense of agency and ownership. No conditions influenced perceptions and attitudes towards self-capacity. Considered together with past literature, the findings provide support for the importance of the embodiment of bodily changes to induce changes in perception. Whether shifts in attitudes towards self-capacity involve different mechanisms or necessitate greater exposure to illusory changes requires further research.

## Supplementary Information

Below is the link to the electronic supplementary material.

Supplementary file1 (DOCX 47 kb)

## Data Availability

The datasets generated during and/or analysed during the current study are available from the corresponding author on reasonable request.
