# Supplementary material for: The effect of visually manipulating back size and morphology on back perception, body ownership, and attitudes towards self-capacity during a lifting task
Source: Psychol Res. 2021 Nov 2;86(6):1816–29. doi: 10.1007/s00426-021-01609-z (PMC9363286; doi:10.1007/s00426-021-01609-z)
Supplement: Supplementary file 1 — Supplementary file1 (DOCX 47 kb) [file 426_2021_1609_MOESM1_ESM.docx]

**Supplementary Material**

**Supplementary Table 1.**

Results of Friedman’s two-way analysis of variance (ANOVA) and post-hoc^a^ tests for study variables by condition

|  | | | | | Post-hoc p-value (adjusted) | | | | | | |
| --- | --- | --- | --- | --- | --- | --- | --- | --- | --- | --- | --- |
| Variable | N^b^ | t | d^f^ | P^c^ | | Strong-Large | Strong- Reshaped | Strong-  Normal | Large- Reshaped | Large-  Normal | Reshaped-Normal |
| Ownership | 24 | 16.47 | 3 | .00 | | .08 | *.04* | *.00* | 1 | 1 | 1 |
| Agency | 24 | 21.80 | 3 | .00 | | *.00* | *.02* | *.03* | 1 | 1 | 1 |
| Feelings towards own back | 24 | .653 | 3 | .88 | | N.A. | N.A. | N.A. | N.A. | N.A. | N.A. |
| Control | 24 | 23.04 | 3 | .00 | | .15 | *.02* | *.01* | 1 | 1 | 1 |

^a^ Pairwise comparisons with Bonferroni correction; significance level p < .05 for all tests and indicated by italics.

^b^ Number of participants

^c^ Asymptotic Significance (2-tailed test).

**Supplementary Table 2.**

Median (and Interquartile range) ratings for attitudes towards self-capacity

| Condition | Perceived strength | Perceived fitness | Confidence in lifting a light box | Confidence in lifting a heavy boxy | Perceived weight of box | Pain intensity |
| --- | --- | --- | --- | --- | --- | --- |
| Normal | 79.0  (61.0-88.25) | 77.5  (61.25-81.88) | 95.5  (92.0-97.88) | 83.0  (72.5-91.75) | 44.0  (21.5-59.25) | 13.5  (4.25-46.75) |
| Reshaped | 74.5  (65.0-85.25) | 75.5  (64.0-85.75) | 96.0  (93.0-99.0) | 81.5  (70.25-92.75) | 48.0  (19.25-61.0) | 14.5  (1.0-40.75) |
| Strong | 79.0  (67.25-87.25) | 77.5  (65.0-84.0) | 93.25  (89.25-97.0) | 81.0  (76.25-93.5) | 49.0  (19.0-60.0) | 20.0  (10.0-34.0) |
| Large | 75.0  (64.0-82.75) | 74.0  (64.63-82.5) | 93.5  (87.0-98.13) | 83.0  (74.25-90.5) | 48.5  (16.25-54.75) | 15.0  (3.0-45.75) |
